# Editorial: Nutrients, Gut Microbiome, and Intestinal Inflammation

**DOI:** 10.3389/fnut.2022.977513

**Published:** 2022-07-15

**Authors:** Fang Yang, Guoxun Chen

**Affiliations:** ^1^School of Laboratory Medicine, Hubei University of Chinese Medicine, Wuhan, China; ^2^Department of Nutrition, University of Tennessee, Knoxville, Knoxville, TN, United States

**Keywords:** gut microbiome, nutrients, phytochemicals, bioactive compounds, probiotics, health

## Introduction

The meticulous skills and interest in lens making of Antonie van Leeuwenhoek eventually extended human observation capability beyond the edge of sight into the world of microbes that he called animalcules (1). The original observation that diarrhea caused by the use of antibiotics in human patients could be treated with intestinal bacteria from healthy donors led scientists to appreciate the roles of gut microbiome in the general health of the host (2). The gut microbiota is the most complex and largest micro-ecosystem of archaea, bacteria, and eukaryotes residing in the lumen, and plays critical roles in growth, metabolism, immunity, environmental adaption, and behavior of the host. The developments of technologies in molecular biology, various omics, and computation allow efficient and accurate analysis of data derived from DNA sequence, metabolites and proteins from the gut microbiota. With the first use of high-throughput 16S rRNA sequencing to characterize fecal bacteria in 1996, microbiota-centric research related to health and diseases has grown tremendously (3).

The establishment of a gut microbiota goes through colonization, succession and replacement, which increases abundance and diversity, and results in a relatively constant α-diversity microbial structure in the host. Bacteria accounts for 99% of the gut microbiota, whereas archaea, fungi, viruses and protists compose the remaining 1%. There are 39 trillion bacteria in the human gut, and their total number of genes is about 150 times that of human genes (4, 5). A dynamic equilibrium relationship of mutual restriction and interdependence has been formed among the gut microbiota, host, and environment. The diversity of mammalian gut microbiota within and among species is influenced by factors such as host phylogeny, genetics, diet, and environment. Therefore, understanding of gut microbiota potentially benefits the disease diagnosis, lifespan, growth, prevention and treatment of chronic diseases. The Research Topic of “*Nutrients, Gut Microbiome, and Intestinal Inflammation*” has attracted interest from the community and captured the complicated interactions among the nutrients, microbiota, and hosts. This Research Topic included 34 papers that explored the effects of nutrients, phytochemicals, bioactive compounds, probiotics and dietary patterns on the growth performance, intestinal health, immunity and chronic diseases *via* regulating the gut microbiome.

## The contribution of gut microbiota to animal growth performance

**Figure 1:**
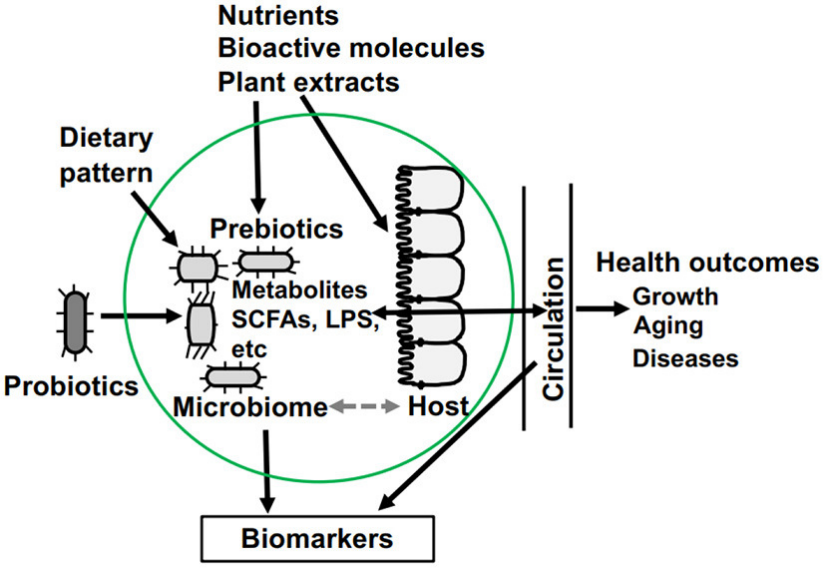
The complex connections among dietary factor, gut microbiota, and hosts. SCFAs, short-chain fatty acids; LPS, lipopolysaccharides.

Animal growth performance, a key part of agriculture, is influenced significantly by the gut microbiota. The papers included in this Research Topic demonstrate that the gut microbiota and dietary factors interact with each other to regulate nutrition, metabolism, immunity, and nervous system in livestock and experimental animals. As shown in Figure 1, the environmental factors such as nutrients, bioactive molecules, plant extracts, probiotics and dietary pattern can affect the microbiota, which in turn alter their impacts on the hosts. In addition, probiotics and dietary pattern can also influence the gut microbiota, and interaction between the host and gut microbiota, which modulates the health outcomes. The metabolites derived from the microbe such as short-chain fatty acids (SCFAs) and lipopolysaccharides (LPS) can enter the host circulation and regulate its functions. Biomarkers from the gut lumen and host circulation can be identified for the purpose of diagnosis. All these affect body weight and feed digestibility, risk of infection and autoimmune diseases, and the body's response to cancer treatment drugs.

The colonization preference of gut microbiota in the gastrointestinal (GI) tract is region-specific and may be determined by changes in physicochemical parameters in the gut lumen, such as pH, nutrient availability, dissolved oxygen, bile acids, antimicrobials, mucins et al. Furthermore, the microbial diversity within a gut segment may vary between the lumen and villus crypts. For example, *Firmicutes* and *Bacteroidetes* predominate in the cecum, while the crypts are mainly colonized by *Proteobacteria* and *Deferribacteres* (6). The *Firmicutes* and *Bacteroidetes* are the most prevalent phylum in ruminant animal's rumens, with *Prevotella* being the most abundant genus. The higher the *Firmicutes* to *Bacteroidetes* (F/B) ratio, the more energy will be obtained from the diet (Shao et al.). In the lactating dairy goats, dietary supplementation of bovine lactoferrin at 200 mg/kg/day for 42 days increased serum lactoferrin levels without affecting feed intake and milk yield, which is associated with the increase in the phylum ratio of F/B and decrease in the abundance of *Prevotella* genus in the rumen (Shao et al.). The results of human clinical trials also suggest that higher caloric intake leads to weight gain *via* the increased F/B ratio (7). On the contrary, caloric restriction may lower the risk of cardiometabolic disease by changing proportions of gut microbiota such as *Akkermansia, Desulfovibrionaceae, Ruminococcaceae* and *Hydrogenoanaerobacterium* (8). *Akkermansia muciniphila* can thicken the intestinal mucus layer, improve metabolism, and facilitate more energy usage in the host, which has become a popular weight loss probiotic, and prebiotics targeting *Akkermansia* may be developed (9). Therefore, to promote livestock growth performance and alleviate obesity and related chronic metabolic diseases in humans, it is helpful to identify specific gut microbiota and promote the establishment of healthy microbial community.

The feed in the rumen is processed by microorganisms and digestive enzymes. The resulted products are used by the microbes and host. SCFAs, such as acetate, propionate, and butyrate from fermentation of dietary polysaccharides by the gut microbiota, and other bioactive ingredients released from the feeds can enter the host body. SCFAs are important energy sources and signaling molecules. Certain gut microbiota such as *Faecalibacterium prausnitzii, Clostridium butyricum, Butyricicoccus pullicaecorum, Roseburia* and *Anaerostipes* ferment dietary fiber, resistant starch, and undigested diets to generate SCFAs (10). The high SCFAs concentrations in the cecum and proximal colon become energy sources for colonocytes and enter the portal vein into the peripheral circulation to act on the liver and peripheral tissues. These SCFAs regulate host metabolism, immune system, and cell proliferation (3). Papers in this Research Topic show that supplementations of odd- and branched-chain fatty acids (Xin et al.), Aspergillus oryzae culture (Guo L. et al.), lauric acid (Wu Y. et al.), coated zinc oxide (Sun Y. et al.), and *Clostridium butyricum* (Li H. et al.) boost the energy supply from SCFAs. Butyrate, one of the SCFAs, has a variety of physiological functions. Using ruminal fluid from male small-tailed Han sheep in an *in vitro* experiment, it has been shown that the increase in sugar to starch ratio leads to elevation of butyrate production without reduction of pH, which is associated with the change of abundance of certain bacterial strains (Dong et al.). It is unclear whether this beneficial effect is produced by butyrate itself and/or in combination with other metabolites produced after fermentation.

In this Research Topic, studies show that dietary supplementation of selenium (Zheng et al.), bovine lactoferricin (Shao et al.), odd- and branched-chain fatty acids (Xin et al.), amylose and amylopectin with different ratios (Gebeyew et al.), barley starch (Ma X. et al.), soluble protein (Zhang et al.), isopropyl ester of 2-hydroxy-4-(methylthio)-butyrate acid (Qin X. et al.), sodium butyrate (Yang et al.), and Aspergillus oryzae culture (Guo L. et al.), benefit growth performance, GI tract health and nutrient digestibility by changing rumen and gut microbiome composition and diversity in ruminants. In addition, supplementation of phospholipids changes the intestinal microbiome, promotes intestinal integrity, and improves growth performance in largemouth bass larvae (Wang et al.). In the fattening Hu sheep, feeding of low-protein diets for 5 weeks reduces the amino acid metabolism pathways, and increases the synthesis of unsaturated fatty acids in the rumen without affecting the growth performance (Zhang et al.).

## The impacts of gut microbiota on aging

The gut microbiota forms along with the birth of an animal and maintains a high degree of consistency throughout adulthood. The analysis of the nature and origin of the gut microbiome community (enterotype) in four pig breeds in China shows significant variations of intestinal micro-environment, which is attributed to the host genotypes, but not dietary patterns (Ma N. et al.). In Holstein cow-calf, the bacteria involved in the digestion are mainly maternally derived (Zhu et al.). However, as one ages, the lifestyles, physiology, immune systems, and properties of guts change, which leads to alterations of gut microbiomes. Aging is associated with changes of microbial diversity, suggesting roles of gut microbiota and microbial metabolites in the lifespan. The specific changes in the microbiota are mainly associated with *Bacteroides, Clostridium, Bifidobacterium* and *Lactobacillus* populations (3).

In this Research Topic, two papers discussed the relationship of aging and gut microbiota. The effects of short-term administration of nicotinamide mononucleotide orally on the fecal microbiota and metabolomes are investigated in pre-aging male mice. Significant reduction of the fecal bacterial diversity with increases in *Helicobacter, Mucispirillum*, and *Faecalibacterium*, and decrease in *Akkermansia* were observed (Niu et al.). The restriction of methionine in the diet for 3 months reshapes the diurnal changes of microbes that cause inflammation, and corrects the integrity of the GI tract in aged (15 months old) male C57BL/6J mice (Ren et al.). This restriction appears to decrease the inflammation-related microbiota (such as *TM7-3, CW040, Staphylococcaceae, Desulfovibrionales*, and *Ruminococcaceae*), and increases the abundance of SCFA-producing microbiota (such as *Prevotella, Bacteroidales, Bacteroides, Lachnospiraceae*, and *Sutterella*) and potential life span-promoting microbiota (such as *Escherichia coli, Akkermansia*, and *Bifidobacterium*) (Ren et al.).

Rumen microbes appear to maintain circadian rhythms that correlate with the melatonin profile in lactating cows (Ouyang et al.). Centenarians and supercentenarians had higher levels of *Christensenella, Akkermansia* and *Bifidobacterium*, indicating potential benefits in longevity (11). It appears that improvement of gut microbiome through dietary interventions, prebiotics and probiotics, and fecal transplant may help to prolong lifespan and slow down aging.

## Gut microbiota and diseases

As a dynamic and critical part of the GI tract, the gut microbiota has been linked to various diseases related to metabolism, GI tract, inflammation, and internal organs. For example, gut microbiota abundance and diversity are inversely associated with obesity, meaning that individuals with low microbiota abundance were at increased risk of obesity, insulin resistance and dyslipidemia, as well as low-grade systemic inflammation (3).

### Chronic metabolic diseases

Obesity, as a chronic metabolic disease, has become a global public health issue. The development of obesity is associated with changes of gut microbiota, and elevated production of LPS and inflammatory responses, which leads to metabolic disturbances (12). Both the diversity of the gut microbiota and ratio of *Bacteroides* to *Firmicutes* in the obese subjects are reduced when compared with that in the lean controls (7). There are two possible mechanisms by which the gut microbiota affects body weight and metabolism in overweight and obese individuals. First, SCFAs derived from fermentation may regulate energy metabolism of the intestinal epithelium and the body. Second, metabolites from gut microbiota such as LPS from Gram-negative bacteria may enter the blood circulation due to weakened tight junctions of the GI tract, and result in immune responses, inflammation, macrophage infiltration and insulin resistance in metabolic active tissues and cells. Dysregulation of the gut microbiota may increase intestinal permeability to gut microorganisms while also increasing the synthesis of toxic microbial metabolites, worsening lipid dysmetabolism and leading to obesity and disorders including diabetes and non-alcoholic fatty liver disease (13). As the gut microbiota plays a role in the control of obesity and metabolism, it has been considered as a therapeutic target for obesity treatment.

Studies included in this Research Topic show that modulation of the gut microbiota can improve obesity and other metabolic diseases. Plant extracts containing active phytochemicals can control obesity *via* the gut microbiota (Weng e al.). The treatment with the instant dark tea (product after the fermentation of *Eurotium Cristatum*) for 12 weeks modulated obesity and lipid metabolism disorder in high fat diet-treated rats by reducing oxidative stress, improving lipid metabolism and glucose metabolism, and enhancing beneficial *Akkermansia* in the gut (Qin S. et al.). Oxidative stress and lipid metabolism were significantly correlated with seven important genera of gut microbiota, which could be potential biomarkers. These genera include *Akkermansia, Clostridiales, Lachnospiraceae, Lachnospiraceae UCG-010, Ruminiclostridium 9, Ruminococaceae-UCG-0*05, and *Ruminocuccus 1* (Qin S. et al.).

The growth in the number of overweight and obese people in the world has led to the development of a metabolic condition known as metabolic syndrome (MetS). The pathogenesis seems to be mostly linked to an imbalance in the redox signaling pathways, gut microbiota, and lipid and glucose metabolism. The benefit of vine tea extract, a product containing significant amount of dihydromyricetin (DHM), has been reviewed and is attributed to raising the relative abundance of *Akkermansia muciniphila* and upregulating the ratio of F/B (Zhou et al.). However, the effects of DHM changing gut microbial composition on gut function, immunity, and inflammatory development are yet to be investigated.

For the glycemia and lipid metabolism, polysaccharides from pumpkin seeds are also able to change the microbiome and reduce blood glucose in mice with type 2 diabetes (Wu H. Q. et al.). The treatment with pumpkin polysaccharides lowered *Erysipelotrichaceae* and increased *Akkermansia* abundance, and reduced the harmful species such as *Clostridium, Thermoanaerobe, Symbiotic bacteria, Deinococcus, Vibrio haematococcus, Proteus gamma*, and *Corio* (Wu H. Q. et al.). The treatment with β-glucan from Baker's yeast, *Saccharomyces cerevisiae*, for 30 days appears to delay the onset of type 1 diabetes (T1D) and suppresses gut inflammation in 2 week-old non-obese diabetic mice, which is attributed to the modulation of the structure and functions of gut microbiota (Taylor and Vasu). T1D is an autoimmune disease leading to disruption of pancreatic β-cells that are responsible for insulin production. In comparison to the control mice, mice treated with β-glucan had increased *Bacteroidetes* abundance and decreased *Firmicutes* and *TM7* levels (Taylor and Vasu).

### Inflammatory bowel disease

Inflammatory bowel disease (IBD) including ulcerative colitis (UC) and Crohn's disease (CD) is an idiopathic intestinal inflammatory disease affecting the ileum, rectum, and colon with clinical manifestations including diarrhea, abdominal pain, and even bloody stools. UC is a continuous inflammation of the colonic mucosa and submucosa, which usually begins from the rectum and gradually spreads to the entire colon. CD can involve the entire digestive tract and is a discontinuous inflammation, especially in the terminal ileum, colon and perianal (14). The development of IBD in humans is associated with changes of diversity and relative abundance of certain gut bacteria, which has practical implications in the disease treatment and diagnosis (Guo X. et al.; Jiang M. et al.). The microbial markers (anti-*Saccharomyces cerevisiae*, ASCA, ASCA IgG, ASCA IgA, anti-mannobioside carbohydrate IgG antibodies, AMCA, anti-chitobioside carbohydrate IgA, ACCA and Anti-I2) from human gut can be used to differentiate CD and intestinal tuberculosis, which share clinically similar symptoms and endoscopic characteristics (Jiang M. et al.). The advantages and disadvantages of utilizing conventional and microbiota biomarkers to evaluate disease severity and treatment results, and possibilities of adopting microbiome-focused therapies during IBD therapy have been discussed in this Research Topic. The relative abundance of *Proteobacteria* in IBD patients is higher than that in the healthy controls, but the relative abundance of *Bacteroides, Eubacterium*, and *Faecalibacterium* are lower (Guo X. et al.). According to the MRI-based radiomic signature, a new nomogram was developed and validated to ease the diagnosis of secondary loss of response to infliximab in CD patients (Feng et al.).

Microbes may be directly used for the treatment of IBD. *Saccharomyces cerevisiae*, a yeast strain, has been engineered to produce lactic acid using glucose, and this strain protects dextran sulfate sodium-induced UC in mice (Sun S. et al.). This probiotic inhibits macrophage pyroptosis, decreases intestinal immune response and modifies the gut microbiota, which is attributed to the regulation of histone modifications by lactate (Sun S. et al.). *Bacillus amyloliquefaciens 40*, a probiotic isolated from Jinhua pig, efficiently ameliorated intestinal structure damages, decreased inflammatory responses, and balanced gut microbiota in mice challenged with *Clostridium perfringens* (Jiang Z. et al.). The pretreatment of *Clostridium butyricum* for 15 days attenuated the *Escherichia coli* K88-induced oxidative damages in the gut and altered the colon microbiome in mice (Li H. et al.). *Clostridium butyricum* can strengthen antioxidant capacity and ameliorate oxidative damages by markedly lowering malondialdehyde (MDA) levels and significantly increasing superoxide dismutase (SOD) and glutathione peroxidase levels in the mouse serum. Furthermore, the SCFA concentrations in the cecum of ETEC K88-infected mice elevated after the enrichment of *Clostridium disporicum* and *Lactobacillus* (Li H. et al.).

## The interactions among environment, host genome and the gut microbiota

Many drugs are derived from natural sources. For example, three of 7 statin drugs approved by the Food and Drug Administration in the US, lovastatin, simvastatin, and pravastatin are identified from fungi (15). Therefore, bioactive compounds are used to prevent diseases and enhance the functions of the human or animal body. The dietary phytochemicals are absorbed differently by the host. Some phytochemicals are fermented and metabolized by bacteria in the colon, which can regulate the gut microbiota structure, and their fermentation metabolites can be absorbed into the body and contribute to anti-oxidation, anti-inflammatory, immune regulation, modulations of glucose and lipid metabolism as shown in the papers of this Research Topic (Jing-Wei et al.; Li L. et al.; Qin S. et al.; Robinson et al.; Weng et al.; Wu H. Q. et al.; Zhao et al.; Zhou et al.). In the pig production practice, phytochemicals are considered an ecologically friendly and safe alternative to antibiotics in the feed to promote animal growth and health. Those phytochemicals influence the animal's energy metabolism, increase anti-oxidant activities, alter gut microbiome and modulate immunity (Li L. et al.). Butyrate, a SCFA, and forskolin, a phytochemical with host defense peptides-inducing, barrier-protective, and anti-inflammatory properties, have the potential to be developed as innovative antibiotic alternatives for disease management and prevention in poultry and probably other species (Robinson et al.).

In addition to pure compounds, extracts from plants including mulberry leaf extract, policosanol, cortical moutan, green tea, honokiol, and capsaicin, can also affect gut microbiome and regulate fuel metabolism in humans (Weng et al.). In lactating Chinese Holstein dairy cows, the supplementation of 30 g/d bamboo leaf extract for 7 weeks is able to increase milk protein, and alter relative abundance of metabolites and microbiome compositions in the milk, which is attributed to the antibacterial activity of extract (Jing-Wei et al.). The relative abundance of *Probacteria* is increased, whereas that of *Firmicutes, Corynebacterium_1, Aerococcus* and *Staphylococcus* in the treated group are decreased compared to that in the control group (Jing-Wei et al.). Furthermore, natural plant essential oils offer strong anti-inflammatory properties. The treatment with lemon essential oil and d-limonene decreased the contents of MDA, myeloperoxidase, and inflammatory factors such as tumor necrosis factor 1α, interleukin 1 (IL-1), and IL-6, and increased the contents of SOD in *Escherichia coli*-infected mice (Zhao et al.).

The interaction between foreign microbes and the host has attracted a significant deal of attention, and the discovery of novel probiotics seems to be the way to enhance the health of humans and animals. *Lactobacillus amylovorus SLZX20-1*, was isolated from the feces of Tibetan weaned piglets as a probiotic, and its treatment for 14 days improved feed intake and changed the composition of intestinal microbes in mice (Shen et al.). The increase of feed intake was attributed to its role in the digestion of starch in the ileum and colon (Shen et al.). Whether those probiotics can be used in humans and what the underlying functional mechanisms remain to be investigated.

The gut microbiota interacts closely with the host mainly through small-molecule metabolites. An integrated metabolome-microbiome technique is developed to investigate the link between a host's metabolism and the microbiota found in the gut. At the molecular level, microbiome research is categorized into three levels, microbial, DNA and mRNA. Corresponding research techniques include culture group, amplicon, metagenome, metavirome, and metatranscriptome (16). In this Research Topic, four papers combine 16S and non-targeting metabolomics technique to analyze the relationship between gut microbiota and host metabolism (Gebeyew et al.; Jing-Wei et al.; Niu et al.; Wu Y. et al.). The results reflect the metabolic state of gut microbiota and discover new directions for microbiology research. Taking bamboo leaf extract supplementation as an example, using Spearman's correlation coefficients, clear correlations between the significantly altered milk microbiota (at the genera level) and metabolite composition were identified. When examining feed additives like bamboo leaf extract for their influences on animal health, milk supply, and quality, the findings illustrate the importance of studying the links between an animal's microbiota and detectable metabolic changes of the host (Jing-Wei et al.). Metagenome combined with microbiome data can screen for key microorganisms that affect specific metabolites.

## Conclusions and future perspectives

The development of technology platforms for DNA/RNA, protein, and metabolite detection and advances in computational techniques have transformed the field of microbial community analysis research. Now, the gut microbiota in the host GI tract can be determined and evaluated after gene sequencing, and the interactions among the host, microbiota and the environmental factors can be analyzed after a combination of metagenome, metabolomics, metatranscriptomics, and metaproteomics and a collection of clinical/environmental data. The papers collected in this Research Topic demonstrated the complex relationships among the gut microbes and environmental factors, and their impacts on the host health and aging. Specific biomarkers and metabolic pathways that promote growth, immunity and health, and disease diagnosis, prevention and treatment have been proposed.

The data show that the host and the environment factors can affect the composition and quantity of the microbiota. On the other hand, the gut microbiota also produces various metabolites with potent signaling functions, such as derivatives of bile acids and amino acids. It should be emphasized that the gut microbiota structure and composition vary widely among species, even within the same species, microbial profiles are often inconsistent due to genetics, age, geolocation, dietary patterns and nutritional composition. It is interesting to note that the nature and origin of the gut microbiome community (enterotype) in pigs is attributed to the genotypes rather than dietary patterns (Ma N. et al.). Therefore, a key goal of the gut microbiota research is to identify a “core microbiome” as a universal microbial taxonomic signature of all individuals that remains largely unchanged.

Just like research progresses in other fields, many unsolved questions remain. Extracts from Chinese herbal medicines and medicinal foods are of great significance for maintaining lipid homeostasis and preventing obesity. However, the regulatory effectiveness of plant extracts from various sources and conditions, the mechanisms of plant extracts affecting gut microbiota, and the anti-obesity benefits of plant extracts in various animals and humans under various metabolic situations can be different. More work should be done to reveal the functions of plant extracts in the host lipid metabolism, so that better dietary strategies can be developed to reduce obesity and maintain the health at individual and community levels.

Another important challenge is to determine the exact role of SCFAs in patho/physiology states of the host and to clarify their mechanisms of actions. The difficulty may lie in the different roles of SCFAs in different tissues, and in different cell types of the same tissue or differentiation states. SCFAs may play a role in many organs, and understanding the spatiotemporal concentrations of metabolites and their functions will help to elucidate the mechanisms by which microbial metabolites affect the host health.

To further analyze the action mechanisms of nutrients, drugs or bioactive compounds in the host through key gut microbiota-metabolites, technology advances in the microbiome, metabolome and target organomics should be integrated and probably co-evolve with artificial intelligent to analyze ever-accumulating big data. Complete understanding of how the experimental conditions affect the composition and functions of the gut microbiota, and then affect the productions of metabolites that act in the target organs of the host will not only benefit human and animal health but also economic development as well. Of course, this is anticipated in the foreseeable future.

## Author contributions

FY discussed the literature and wrote the manuscript. GC designed the outline, edited, and revised the manuscript. Both authors approved the final version of this editorial.

## Funding

This work was supported by the Scientific Research Plan of Hubei Provincial Health Commission (No. WJ2021Q050).

## Conflict of interest

The authors declare that the research was conducted in the absence of any commercial or financial relationships that could be construed as a potential conflict of interest.

## Publisher's note

All claims expressed in this article are solely those of the authors and do not necessarily represent those of their affiliated organizations, or those of the publisher, the editors and the reviewers. Any product that may be evaluated in this article, or claim that may be made by its manufacturer, is not guaranteed or endorsed by the publisher.
